# Effect of New Zealand Blackcurrant Extract on Physiological Responses at Rest and during Brisk Walking in Southeast Asian Men: A Randomized, Double-Blind, Placebo-Controlled, Crossover Study

**DOI:** 10.3390/nu10111732

**Published:** 2018-11-12

**Authors:** Mark Elisabeth Theodorus Willems, Nisakorn Parktin, Waree Widjaja, Amornpan Ajjimaporn

**Affiliations:** 1 University of Chichester, Institute of Sport, College Lane, PO19 6PE Chichester, UK; 2 College of Sports Science and Technology, Mahidol University, Salaya, Nakhon Pathom 73170, Thailand; n.parktin@gmail.com (N.P.); widjaja.waree@gmail.com (W.W.); g4036011@gmail.com (A.A.)

**Keywords:** anthocyanins, sports nutrition, health promotion, substrate oxidation, cardiovascular function, ethnicity, indirect calorimetry

## Abstract

New Zealand blackcurrant (NZBC) extract affects cardiovascular and metabolic responses during rest and exercise in Caucasian men. Ethnicity and nutritional habits may affect responses to nutritional ergogenic aids. We examined the effects of NZBC extract on cardiovascular, metabolic, and physiological responses during seated rest and moderate-intensity exercise in Southeast Asian men. Seventeen healthy Thai men (age: 22 ± 3 years; body mass index (BMI): 21.8 ± 1.1 kg·m^−2^) participated. Resting metabolic equivalent (1-MET) was measured (Oxycon™ mobile, Germany), and an incremental walking protocol was completed to establish the relationship between walking speed and MET. In a double-blind, randomized, placebo-controlled, crossover design, cardiovascular (Physioflow, *n* = 12) and physiological responses (Oxycon, *n* = 17) were measured during both seated rest and a 30-min treadmill walk at five metabolic equivalent (5-MET), with either a seven-day intake of placebo (PL) or two capsules of NZBC extract (each 300 mg capsule contains 35% blackcurrant extract) with a 14-day washout. Paired *t*-tests were used with significance accepted at *p* < 0.05 and a trend for 0.05 > *p* ≤ 0.10. During 30 min of treadmill walking at 5-MET, no differences were observed for heart rate and substrate oxidation. With intake of NZBC during treadmill walking, there was a trend for increased stroke volume by 12% (PL: 83.2 ± 25.1; NZBC: 93.0 ± 24.3 mL; *p* = 0.072) and cardiac output increased by 12% (PL: 9.2 ± 2.6; NZBC: 10.3 ± 2.8 L·min^−1^; *p* = 0.057). Systemic vascular resistance decreased by 10% (PL: 779 ± 267; NZBC: 697 ± 245 dyn·s·cm^−5^; *p* = 0.048). NZBC extract had no effect on metabolic, physiological, and cardiovascular parameters during seated rest and exercise-induced fat oxidation in Thai men, in contrast to observations in Caucasian men. During treadmill walking, Thai men showed cardiovascular response, indicating vasodilatory effects during moderate-intensity exercise with the intake of NZBC extract. Our findings suggest that the ergogenic responses to anthocyanin intake from New Zealand blackcurrant may be ethnicity-dependent.

## 1. Introduction

The effectiveness of popular sport nutrition supplements for exercise is underpinned primarily by observations from experimental studies with Caucasian participants (e.g., beetroot juice [1] and sodium bicarbonate [2]). Ethnicity, however, may affect physiological, metabolic, and cardiovascular responses to nutritional interventions. For example, with caffeine intake, the response in cytochrome P450 1A2 activity was different in South Asians compared to Caucasians [3]. Overall, there is a dearth of information on the effectiveness of sport nutrition supplements in non-Caucasians. In the last few years, supplements with polyphenol composition (e.g., green tea, pomegranate, blueberry, Montmorency tart cherry, chokeberry, and blackcurrant) have emerged as sport nutrition supplements, due to potential antioxidants and associated beneficial health effects, with observations primarily in Caucasians.

In the case of blackcurrant, the numerous health benefits [4] are due to the specific anthocyanin content, consisting primarily of delphinidin-3-rutinoside, delphinidin-3-glucoside, cyanidin-3-rutinoside, and cyanidin-3-glucoside [5]. Intake of one of the blackcurrant anthocyanins (i.e., cyanidin-3-glucoside) results in numerous metabolites with dynamic appearance profiles [6], large inter-individual recovery rates in humans [7], and metabolites associated with the alteration of cell function [8]. New Zealand blackcurrant extract enhances whole-body, exercise-induced fat oxidation in endurance-trained men [9]. Blackberry, a berry with a high content of primarily cyanidin-3-glucoside, also increases fat oxidation at rest and during a 30-min treadmill walk in overweight and obese males with seven-day intake who are being fed a high-fat diet [10]. In contrast, Montmorency tart cherry juice intake for 20 days did not affect fat oxidation during exercise or rest [11]. With respect to cardiovascular responses, a larger femoral artery diameter by about 6–8% was observed during a two-minute, 30% isometric contraction of the knee extensors in males with blackcurrant intake [12]. In Cook et al. [12], blackcurrant had no effect on heart rate at rest and during the 30% isometric contraction of the knee extensors. However, during the 30% isometric contraction, systolic blood pressure and total peripheral resistance were reduced while cardiac output increased with blackcurrant intake [12]. Such observations by Cook et al. [12] indicate a change in blood flow and confirm an earlier finding of an increase in peripheral blood flow with blackcurrant intake by Matsumoto et al. [13]. Intake of Montmorency tart cherry lowered blood pressure at rest, and the improvements in cardiovascular risk factors were associated with the metabolites vanillic acid and protocatechuic acid [14]. Protocatechuic acid and vanillic acid also appear in the blood with intake of the blackcurrant anthocyanin cyanidin-3-glucoside [6,7]. In-vitro studies have shown effects on human vascular cells with cyanidin-3-glucoside-induced metabolites [8,15], endothelial function in coronary arterial rings with chokeberry extracts [16], and human umbilical vein endothelial cells with blackberry juice [17]. Chokeberry juice intake lowered blood pressure in people with mild hypertension [18]. However, we are not aware of other high-anthocyanin-containing supplements other than New Zealand blackcurrant with effects on both metabolic and cardiovascular responses at rest as well as during exercise. Evidence about the beneficial effects of anthocyanin intake via different berries by observations from in-vitro and in-vivo studies is growing; for recent reviews regarding health effects, see Fang [19], Cerletti et al. [20] and Cassidy [21], plus Cook and Willems [22] for physiological responses and performance effects.

For polyphenol supplementation, it has been suggested that the gut microbiome plays an important role in the bioavailability of metabolites and the interindividual differences in bioavailability [23]. The bioavailability of anthocyanins in blood plasma is very low. The anthocyanin-induced appearance of metabolites is affected by absorption, distribution, and conversion of ingested anthocyanins. Dietary habits affect the gut microbiome [24]. Dietary habits are influenced by many factors, including geographical location, ethnicity [25], and food availability. Lower utilization of lipids in Thai men during exercise was suggested to be due to dietary habits [26]. In sports and exercise nutrition research, it is recognized that the effectiveness of supplements needs to be established with consideration of sex, age, and training status, whereas ethnicity and dietary habits seems to be ignored. No studies have examined the effect of a nutritional ergogenic aid on substrate oxidation at rest and during exercise in non-Caucasians. Enhanced fat oxidation at rest and during moderate-intensity exercise is considered beneficial; it could provide health benefits, and is affected by nutritional ergogenic aids.

Therefore, the primary aim of the present study is to examine the effect of New Zealand blackcurrant extract on the cardiovascular (i.e., heart rate, stroke volume, cardiac output, and systemic vascular resistance), metabolic (i.e., substrate oxidation and respiratory exchange ratio), and physiological (i.e., minute ventilation and oxygen uptake) responses at rest and during a 30-min brisk treadmill walk in Southeast Asian men. In our work with Caucasian male participants, we have observed beneficial effects on cardiovascular function at rest and during submaximal isometric contraction (e.g., increased cardiac output), as well as enhanced whole-body fat oxidation during cycling. For the present study, it was hypothesised that New Zealand blackcurrant extract will increase cardiac output at rest and increase fat oxidation during the brisk treadmill walk. It was observed by Robinson et al. [27] in Caucasians that resting fat oxidation correlated significantly with exercise-induced maximal fat oxidation. Therefore, the second aim of the study was to examine whether such a significant correlation exists between whole-body fat oxidation at rest and during brisk walking in Southeast Asian men.

## 2. Materials and Methods

### 2.1. Participants

Seventeen healthy Southeast Asian (i.e., Thai) men from a university population (age: 22 ± 3 years; height: 172 ± 5 cm; body mass: 64.3 ± 5.0 kg; body fat: 15 ± 4%; systolic blood pressure: 107 ± 37 mm Hg; diastolic blood pressure: 70 ± 7 mmHg; body mass index (BMI): 21.8 ± 1.1 kg·m^−2^ (mean ± standard deviation (SD)); range BMI: 19.5–23.4), sixteen with normal weight and one overweight [28], volunteered for the study. Participants received cash payment from Mahidol University and provided written informed consent after explanation of the experimental procedures, potential risks, and benefits of the study. Participants were non-smokers, recreationally active, and did not take dietary or sports nutrition supplements. Ethical approval was obtained from Mahidol University (MU-CIRB 2017/115.1906), with protocol and procedures conforming to the 2013 Declaration of Helsinki.

### 2.2. Experimental Design

The study was a double-blind, randomized, placebo-controlled crossover design. Randomization was achieved by flipping a coin. Participants visited the air-conditioned exercise physiology laboratory (temperature: 25.2 ± 0.5 °C; humidity: 45 ± 4%; barometric pressure: 1008 ± 2 mbar; mean ± SD) on three occasions in the morning, over a 5–6 week period. In preparation for all laboratory visits and testing sessions, participants were instructed to abstain from strenuous exercise for 48 h, not consume alcohol for 24 h before testing, and consume no caffeine-containing products on the day of testing.

During the first visit, height (Nagata S/N CH2882, Taiwan), body mass, and body fat were measured with bioelectrical impedance analysis (Omron HBF-375, Kyoto, Japan). Blood pressure at rest was taken two times using an automatic blood pressure monitor (Omron SEM-1, Kyoto, Japan). Then, participants were seated for 10 min in the laboratory with the lights dimmed, and 2 × 10 min expired air collections were measured separated by five minutes, to determine the individual oxygen consumption at rest (i.e., the metabolic equivalent, 1-MET [29]) (Oxycon™ mobile, CareFusion, Germany). The lowest value of oxygen consumption during the 2 × 10 min expired air collections was taken as the 1-MET, i.e., 4.04 ± 0.44 mL·kg^−1^·min^−1^ (mean ± SD) (range 1-MET: 3.23–4.62 mL·kg^−1^·min^−1^). During the seated rest, participants were familiarized with the setup for cardiovascular recordings (Physioflow^®^, Bristol, PA, USA), and fitted with skin electrodes (Ambu^®^, Bluesensor, Malaysia) according to manufacturer guidelines. In the same session, participants completed an incremental-intensity walking protocol on a treadmill (Marquette Series 2000 treadmill, Waukesha WI, United States) with stages of 8 min. Walking speed at the start was 2 km·h^−1^, with stage increments of 1 km·h^−1^. Normally, five or six stages of 8 min were completed to achieve oxygen consumption close to or over five metabolic equivalent (5-MET). During each eight-minute stage, expired air was analysed for the last 3 min. The incremental-intensity walking protocol was performed to determine the linear relationship between walking speed and oxygen consumption [30], with the oxygen consumption expressed as the corresponding metabolic equivalent. The linear relationship between walking speed and metabolic equivalent (*r*^2^ = 0.9364 ± 0.0363, mean ± SD) was used to calculate the walking speed at 5-METs (i.e., moderate intensity exercise) for visits 2 and 3.

Before attending the laboratory for visits 2 and 3, participants consumed two capsules per day of New Zealand blackcurrant (NZBC) extract (CurraNZ, Health Currancy Ltd., Surrey, United Kingdom) or placebo for seven days. Each NZBC extract capsule of 300 mg contained 105 mg of anthocyanins, i.e., 35–50% delphinidin-3-rutinoside, 5–20% delphinidin-3-glucoside, 30–45% cyanidin-3-rutinoside, and 3–10% cyanidin-3 glucoside, with the remaining content mainly natural plant sugars. One capsule equals about 85 New Zealand blackcurrants. Composition information was provided by Health Currancy Ltd., Surrey, UK. The placebo was an identical-looking capsule, each capsule containing 300 mg of microcrystalline cellulose M102. For six days, one capsule was taken with breakfast and one with dinner. Participants were advised to take the last two capsules of the blackcurrant extract or placebo with a standard breakfast of one slice of bread and water two hours before attending the laboratory for the testing sessions. The wash-out period was 14 days. A 14-day wash-out period has been found to clear anthocyanins out of the blood [31]. The optimal dosing strategy for NZBC extract is not known. However, previous studies have used a similar dosing strategy for the duration and amount of NZBC extract and wash-out period [12,32]. In the second and third visit, expired air and cardiovascular responses were collected during a 2 × 10 min period and during 30 min of treadmill walking at 5-MET (5-MET walking speed: 6.1 ± 0.5 km·h^−1^’ range: 4.8–7.2 km·h^−1^). Physioflow recordings of five participants were erratic and therefore not analysed.

Participants recorded their dietary intake for seven days prior to the first experimental condition (i.e., visit 2), and were instructed for the subsequent experimental visit (i.e., visit 3) to replicate their intake. Food diaries were analysed with INMUcal v.3 NB.3, with nutritive values for Thai food (Institute of Nutrition, Mahidol University, Salaya, Nakhon Pathom, Thailand) for carbohydrate, fat, and protein intake, as well as total energy intake (kJ) (Table 1).

The respiratory exchange ratio (i.e., RER) was calculated by the ratio of the volume of carbon dioxide produced and volume of oxygen consumption. Rates of whole-body fat and carbohydrate oxidation were calculated with equations 1 and 2 from Jeukendrup and Wallis [33], based on the stoichiometry of glucose and palmitic acid and the assumption of negligible protein oxidation:

(1) $$Fat\;oxidation\left(g\cdot min^{-1}\right)=1.695\times \dot{V}O_{2}-1.701\times \dot{V}CO_{2}$$

(2) $$Carbohydrate\;oxidation\left(g\cdot min^{-1}\right)=4.210\times \dot{V}CO_{2}-2.962\times \dot{V}O_{2}$$

### 2.3. Statistical Analysis

Statistical analyses were completed using GraphPad Prism (version 5 for Windows; GraphPad Software, La Jolla, CA, USA). Data normality assumptions were tested with the Kolmogorov–Smirnov test, with minute ventilation at rest not passing normality. Based on the hypothesis that NZBC extract would result in a change for metabolic, physiological, and cardiovascular observations in studies with sample sizes equal to or less than fifteen [9,12,32], one-tailed paired sample *t*-tests used were to compare all of the parameters between the placebo and NZBC extract conditions, performing a Mann–Whitney U test for minute ventilation at rest. Effect sizes (i.e., Cohen’s *d* [34]) were calculated for changes that were significant or showed a trend for significance, with an effect size of <0.2 as a trivial, 0.2–0.39 as a small, 0.4–0.69 as a moderate, and ≥0.7 as a large magnitude of change. Pearson correlation coefficients were calculated for the relationship between fat oxidation at rest and during the 5-MET 30-min walk for the placebo and NZBC extract conditions. Statistical significance was accepted at *p* < 0.05. The interpretation of *p* as 0.05 > *p* ≤ 0.1 was according to guidelines by Curran-Everett and Benos [35]. Data are presented as mean ± SD.

## 3. Results

### 3.1. Physiological and Metabolic Observations at Rest 

At rest, New Zealand blackcurrant had no effect on minute ventilation (PL: 8.60 ± 1.53; NZBC extract: 8.70 ± 1.76 L·min^−1^; *p* = 0.49), oxygen uptake (PL: 4.29 ± 0.66; NZBC extract: 4.38 ± 0.72 mL·kg^−1^·min^−1^; *p* = 0.29), RER (PL: 0.86 ± 0.05; NZBC extract: 0.86 ± 0.06; *p* = 0.40), carbohydrate oxidation (PL: 0.19 ± 0.07; NZBC extract: 0.18 ± 0.06 g·min^−1^; *p* = 0.37), or fat oxidation (PL: 0.06 ± 0.03; NZBC extract: 0.07 ± 0.04 g·min^−1^; *p* = 0.32). An absence of change in fat oxidation at rest may suggest that lipolysis is not affected by blackcurrant intake in Southeast Asian males.

### 3.2. Cardiovascular Function at Rest

At rest, New Zealand blackcurrant had no effect on heart rate (PL: 59 ± 10; NZBC extract: 58 ± 9 beats·min^−1^; *p* = 0.28), stroke volume (PL: 77 ± 19; NZBC extract: 81 ± 21 mL; *p* = 0.29), cardiac output (PL: 4.49 ± 0.99; NZBC extract: 4.42 ± 0.63 L·min^−1^; *p* = 0.43), or systemic vascular resistance (PL: 1509 ± 333; NZBC extract: 1535 ± 251 dynes·s·cm^−5^; *p* =0.43). An absence of any effect on cardiovascular parameters at rest seems to suggest that endothelial function at rest is not affected by blackcurrant intake in Southeast Asian males.

### 3.3. Metabolic and Physiological Responses during the 30-min Treadmill Walk at 5-MET

During the 30-min walk at 5-MET, New Zealand blackcurrant had no effect on minute ventilation (PL: 35.7 ± 6.0; NZBC extract: 35.2 ± 5.6 L·min^−1^; *p* = 0.24), oxygen uptake (PL: 20.91 ± 2.44; NZBC extract: 20.42 ± 3.07 mL·kg^−1^·min^−1^; *p* = 0.16), RER (PL: 0.92 ± 0.06; NZBC extract: 0.91 ± 0.04; *p* = 0.42), carbohydrate oxidation (PL: 1.21 ± 0.37; NZBC extract: 1.16 ± 0.28 g·min^−1^; *p* = 0.26), or fat oxidation (PL: 0.18 ± 0.13; NZBC extract: 0.19 ± 0.10 g·min^−1^; *p* = 0.40). The absence of any changes in exercise-induced fat oxidation suggests that lipolysis and all cellular events in skeletal muscle that contribute to fat oxidation were not affected by blackcurrant intake in Southeast Asian males.

**Figure 1:**
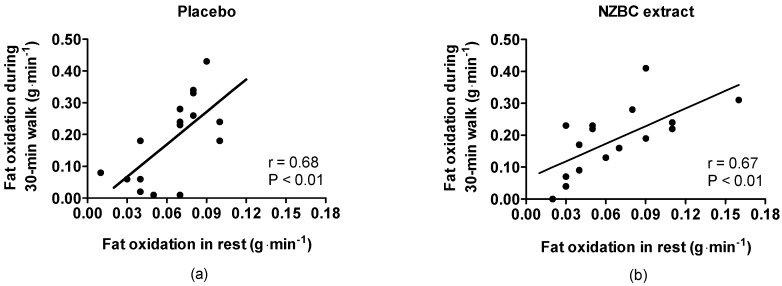
Relationship between whole-body fat oxidation at rest and during a five metabolic equivalent walk for 30 min (**a**) with a placebo and (**b**) under New Zealand blackcurrant (NZBC) extract conditions. The Pearson correlation coefficient was significant in both conditions.

Fat oxidation during the 30-min walk was significantly correlated to both the placebo and NZBC extract conditions with the fat oxidation at rest (Figure 1).

### 3.4. Cardiovascular Responses during the 30-min Treadmill Walk at 5-MET

**Figure 2:**
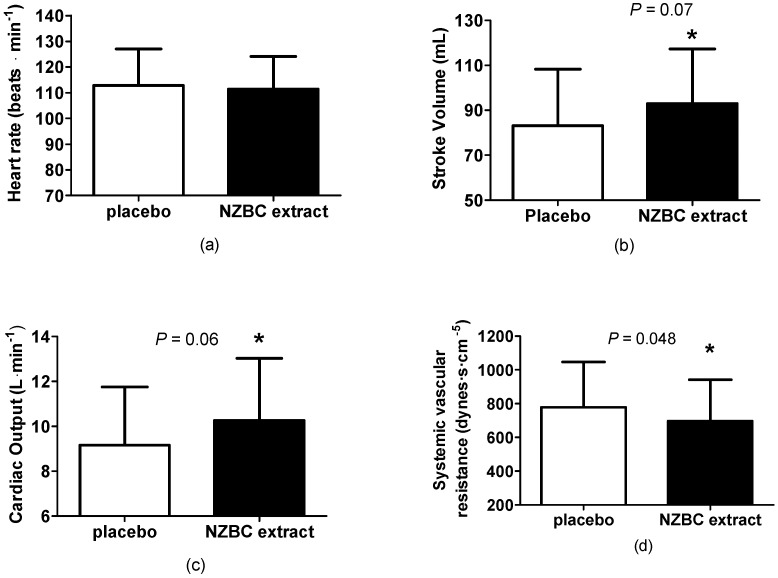
(**a**) Heart rate, (**b**) stroke, (**c**) cardiac output, and (**d**) systemic vascular resistance for the placebo and New Zealand blackcurrant extract during a 30-min treadmill walk at 5-MET. * indicates a difference or a trend for a difference between the placebo and NZBC extract conditions.

During the 30-min walk at 5-MET, New Zealand blackcurrant had no effect on heart rate (PL: 113 ± 14; NZBC extract: 112 ± 13 beats·min^−1^; *p* = 0.27) (Figure 2a), but there was a trend for a larger stroke volume by 12% with a moderate effect size (PL: 83 ± 25; NZBC extract: 93 ± 24 mL; *p* = 0.07, *d* = 0.45, Figure 2b).

There was a trend for larger cardiac output by 12%, with a moderate effect size (PL: 9.15 ± 2.59; NZBC extract: 10.26 ± 2.78 L·min^−1^; *p* = 0.06, *d* = 0.41) (Figure 2c). There was also a significant lower systemic vascular resistance by 11%, with a small effect size. (PL: 779 ± 269; NZBC extract: 697 ± 245 dynes·s·cm^−5^; *p* = 0.48, *d* = 0.32) (Figure 2d). In Southeast Asian males, blackcurrant intake may affect exercise-induced endothelial function and be indicative of additional vasodilation during walking.

## 4. Discussion

We observed in Southeast Asian men, in both the placebo and blackcurrant conditions, that whole-body fat oxidation at rest and during a 30-min brisk walk had significant correlation coefficients of 0.68 and 0.67. As far as we know, the study by Robinson et al. [27] reported for the first time that whole-body fat oxidation at rest and exercise-induced maximal fat oxidation were significantly correlated (*r* = 0.55) in healthy, recreationally active Caucasian men (*n* = 57). Our value for whole-body fat oxidation at rest (0.06 ± 0.03 g·min^−1^) was somewhat lower than that of Robinson et al. [27] (0.08 ± 0.03 g·min^−1^). For exercise, the difference in expressing the exercise intensities in the Robinson et al. [27] and the present study does not allow for study comparison of the values for exercise-induced fat oxidation. In the present study, the values for RER at rest are comparable with those in Thai men in a study by Janyacharoen et al. [26]—e.g., RER at rest (our study: 0.86 ± 0.05; Janyacharoen et al. [26]: 0.86 ± 0.02). Therefore, from our observations and the study by Robinson et al. [27], healthy, recreationally active Southeast Asian and Caucasian men have a similar relationship between fat oxidation at rest and exercise-induced fat oxidation. Exercise-induced fat oxidation, as observed in our study, was low in Southeast Asian men and confirms the observations by Janyacharoen et al. [26]. In addition, Hall et al. [36] also observed that South Asians had reduced fat oxidation during submaximal exercise at the same relative exercise intensity than BMI-matched Caucasian men. Therefore, the absolute values for whole-body fat oxidation at rest and during brisk walking in Thai men in the present study confirm findings in the literature for ethnic differences in substrate oxidation at rest and during exercise.

In general, whole-body fat oxidation requires the mobilization of free fatty acids following triglyceride lipolysis in adipocytes, the transport of free fatty acids in the blood, transfer across cell membranes (e.g., muscle cells), transport across mitochondrial membranes, and beta-oxidation. Peak whole-body fat oxidation seems to be related to lean body mass and maximal oxygen uptake [37]. In addition, a study by Fletcher et al. [38] noted the role of dietary intake being independently associated with maximal fat oxidation during exercise. With respect to beta-oxidation, a role for phytochemicals has been recognized [39]. Whole-body fat oxidation at rest and during brisk treadmill walking was not enhanced with the intake of New Zealand blackcurrant extract in Thai men. As far as we know, only blackberry has been reported to enhance whole-body fat oxidation at rest in males, but ethnicity was not reported [10]. In Caucasian endurance-trained men and women, enhanced exercise-induced fat oxidation was observed with the intake of New Zealand blackcurrant extract [9,32,40]. Interestingly, in the study with Caucasian women [40], the exercise-induced fat oxidation was associated with increased non-esterified fatty acids concentration at rest, an indication of enhanced lipolysis. Therefore, in Southeast Asian men, New Zealand blackcurrant does not seem to affect lipolysis; fat oxidation at rest was also not affected. In addition, it is also possible that a difference in body composition between Thai men in the present study (i.e., BMI: 21.8 ± 1.1 kg·m^−2^) and training status in [9,32] may have been confounding factors. Interestingly, 4-week capsinoid intake in Northeast Asians enhanced fat oxidation more in those with high BMI (>25 kg·m^−2^) [41]. However, Asian men with a similar BMI to Caucasian men had higher body fat [42]. Future studies may want to address the role of body fat on the responsiveness of individuals of different ethnicities on whole-body fat oxidation by the intake of New Zealand blackcurrant. In general, the mechanisms for enhanced whole-body fat oxidation by the intake of anthocyanin-rich supplements are not known. It can also not be excluded that the dietary habits by Thai men may result in a different gut microbiome than Caucasians. Differences in gut microbiome by ethnicity [25] may affect plasma metabolite availability by anthocyanin intake. It is possible that the bioavailability of anthocyanin-induced metabolites in Southeast Asian males was too low to allow changes to any of the cellular events that contribute to whole-body fat oxidation. Therefore, we expect that future studies will address causal relationships for anthocyanin-induced plasma metabolite bioavailability and enhanced whole-body fat oxidation. Such studies may also address whether habitual anthocyanin intake with the normal diet affects the response by supplementation. In the present study, habitual anthocyanin intake was not quantified. Therefore, a limitation of the present study is that the habitual dietary intake of polyphenols limited the potential effects of New Zealand blackcurrant intake on whole-body fat oxidation. In general, enhanced whole-body fat oxidation by exercise would have been considered beneficial. Interestingly, maximal fat oxidation was positively associated with 24-h fat oxidation and insulin sensitivity in Caucasian men [43]. We have shown an increase in insulin sensitivity with 7-day intake of New Zealand blackcurrant powder [44]. In the absence of an enhancement of whole-body fat oxidation in Southeast Asian men in our study with intake of New Zealand blackcurrant, future work may address the response of New Zealand blackcurrant intake in South Asians on insulin sensitivity. In general, South Asians are more insulin resistant than Caucasians [36], so our observations may suggest the South Asians are less responsive to polyphenol intake in their normal diets, compared to Caucasians.

We observed in a previous study with Caucasian men an effect of New Zealand blackcurrant extract on cardiovascular function at rest, i.e., an increased cardiac output and reduced total peripheral resistance [45]. Such changes in cardiovascular function at rest were absent in the present study with Thai men. This would suggest that the intake of New Zealand blackcurrant in the present study did not result in vasodilation at rest. However, in Caucasian men, during a 2-min sustained 30% submaximal isometric contraction of the quadriceps muscles, we observed changes in cardiovascular responses, i.e., increased cardiac output and a decrease in total peripheral resistance with the intake of New Zealand blackcurrant [12]. Such observations were associated with an increase in the diameter of the femoral artery, suggesting enhanced blood flow due to the intake of New Zealand blackcurrant [12]. In the present study, during brisk walking in Thai men, New Zealand blackcurrant affected the cardiovascular response, as evidenced by a trend for enhanced cardiac output and significant decrease in systemic vascular resistance. It seems that in both low static activity in Caucasian men and moderate-intensity exercise in Thai men, New Zealand blackcurrant seems to affect the production of nitric oxide. It is possible that enhanced cardiac output during brisk walking was due to greater venous return. However, there is an inconsistency in the effects of the intake of New Zealand blackcurrant for cardiovascular responses at rest in Southeast Asians and Caucasians. It is possible that the bioavailability of anthocyanin-induced metabolites with the blackcurrant intake needed to change the cell function necessary for vasodilation in Southeast Asians is too low with only a 7-day intake, for changing cardiovascular function at rest, but is sufficient to alter responses during exercise. Future work may want to examine the effects of a higher intake or intake for a longer period of anthocyanin-rich supplements on metabolic and cardiovascular responses at rest and during exercise in Southeast Asians. A limitation of the present study was that no information was available on the anthocyanin-induced metabolites with a 7-day intake of New Zealand blackcurrant.

## 5. Conclusions

It is concluded that the intake of New Zealand blackcurrant extract has no effect on metabolic, physiological, and cardiovascular responses at rest in Thai men with normal body weight. However, New Zealand blackcurrant extract affected cardiovascular response during brisk walking in Thai men. Supplementation studies may need to consider the ethnicity of the study population to examine the effects of nutritional ergogenic aids.

## Figures and Tables

**Table 1:** Daily dietary intake, with absolute and relative to body mass values.

Carbohydrate	(g)	1.68 ± 49
	(g·kg body mass^−1^)	2.62 ± 0.78
Protein	(g)	57 ± 13
	(g·kg body mass^−1^)	0.89 ± 0.23
Fat	(g)	68 ± 14
	(g·kg body mass^−1^)	1.07 ± 0.23
Total energy intake	(kJ)	6087 ± 1293
	(kJ·body mass^−1^)	95 ± 22

Values are the mean ± SD for 17 male participants (age: 22 ± 3 years; BMI: 21.8 ± 1.1 kg·m^−2^).
